# Tightly Coupled GNSS/INS Integration with Robust Sequential Kalman Filter for Accurate Vehicular Navigation

**DOI:** 10.3390/s20020561

**Published:** 2020-01-20

**Authors:** Yi Dong, Dingjie Wang, Liang Zhang, Qingsong Li, Jie Wu

**Affiliations:** 1 College of Aerospace Science and Engineering, National University of Defense Technology, Changsha 410073, China; dongyi13@nudt.edu.cn (Y.D.); liqingsong10@nudt.edu.cn (Q.L.); 2 College of Information and Navigation, Air Force Engineering University, Xi’an 710077, China; zhangliang_nudt@nudt.edu.cn

**Keywords:** GNSS/INS, tightly coupled integration, robust KF, sequential KF, computational efficiency, vehicular navigation

## Abstract

With the development of multi-constellation multi-frequency Global Navigation Satellite Systems (GNSS), more and more observations are available for tightly coupled GNSS/Inertial Navigation System (INS) integration. Concerning the accuracy, robustness, and computational burden issues in the integration, we proposed a robust and computationally efficient implementation. The new tight integration model uses pseudorange, Doppler and carrier phase simultaneously, to achieve the maximum possible navigation accuracy for a single receiver. The resultant high-dimensional observation vector is then processed by a sequential Kalman Filter (KF) to improve the computational efficiency in the measurement update step. Based on the innovation of the sequential KF, a robust estimation method with Gaussian test is further devised to detect and adapt the faults in individual GNSS channels. Two field vehicular tests are conducted to evaluate the performance improvements of the proposed method, compared with loose coupling and conventional tight coupling. Test results in favorable environments indicate that the proposed method can significantly improve the velocity and attitude accuracy by 69.42% and 47.16% over loose coupling and by 64.75% and 30.88% over conventional tight coupling, respectively. Moreover, the computational efficiency is also improved by about 53.09% for the proposed method, compared with batch KF processing. In GNSS challenging environments, the proposed method also shows superiority in terms of velocity and attitude accuracy, and better bridging capability during the GNSS partial or complete outages. These results demonstrate that the proposed method is able to provide a more robust and accurate solution in real-time vehicular navigation.

## 1. Introduction

Accurate and reliable navigation information is a fundamental basis of many vehicular applications such as autonomous driving, location based services, and traffic management. With global coverage, all-weather capability, and good long-term accuracy, Global Navigation Satellite Systems (GNSS), such as the U.S. Global Positioning System (GPS), the Chinese BeiDou navigation satellite system (BDS), Russian GLObal NAvigation Satellite System (GLONASS), and European Galileo, are widely utilized in current vehicle navigation systems. However, using GNSS alone has certain limitations. First, the satellite visibility is completely or partially obscured in degraded environments such as urban canyons, tunnels, and buildings. Second, the GNSS signal is vulnerable to interference such as spoofing and jamming [1]. Last, GNSS cannot provide full vehicle states including position, velocity, attitude, and angular rate, which are needed in the control systems [2]. It is therefore necessary to introduce augmented sensors to enhance the overall performance.

Owing to its autonomy, high update rate and considerable short-term accuracy, Inertial Navigation System (INS) is a mutually complementary mate for GNSS [3]. GNSS/INS integration can achieve more accurate and robust navigation than either stand-alone system. Especially, the great advances in low-cost GNSS receivers and Micro-Electro-Mechanical Systems (MEMS) have dramatically reduced the hardware cost for GNSS/INS integration, making it popular in vehicular navigation. At present, loose coupling (LC) and tight coupling (TC) are two well-developed integration strategies [4]. LC fuses GNSS-derived position and velocity with INS. LC is simple in implementation and requires the least amount of GNSS knowledge. The disadvantages are that the computation of position and velocity is not feasible with less than four satellites, and if this computation in GNSS engine is implemented in a filtering way, cascade filtering problem in GNSS/INS integration will arise.

To avoid the above drawbacks, TC is preferable. TC directly uses GNSS raw observations for measurement update, thus continuous aiding is feasible even with fewer than four satellites. Consequently, TC performs better in GNSS partly blocked areas. Conventional TC adopts pseudorange and Doppler observations for state estimation (TC-PD), which is simple and applicable in a single receiver case. However, due to the high noise and multipath error, the position accuracy is comparable to single point positioning (SPP). Unlike pseudorange, carrier phase is less effected by multipath and the multipath error is generally 100 times smaller (centimeter level) [5]. Carrier phase is able to achieve centimeter level positioning accuracy when successfully solving the ambiguities. Therefore, many researches have been concentrated on integrating carrier phase with INS in TC, including real time kinematic (RTK)/INS [6,7,8] and precise point positioning (PPP)/INS [9,10,11]. RTK requires aiding of the base station to fix the ambiguity parameters, which is not feasible in situations where the base station is not available or affordable. PPP can work in a single receiver case with precise orbit and clock products provided by a global reference network (e.g., the International GNSS Service network). However, it generally requires a comparatively long time to become (re)convergent, which is not tolerable in some real-time applications. To exploit the high accuracy of carrier phase for a single receiver in real-time navigation, time-differenced carrier phase (TDCP)/INS integration is developed [12,13,14,15]. The bothersome ambiguities are eliminated in TDCP since ambiguities remain constant if no cycle slips occur. In addition, the temporal correlated ionospheric and tronospheric delays are also mitigated for a short sampling interval, which further benefits the accuracy of carrier phase [12,13].

Apart from accuracy, the reliability of observations is also an important concern in TC. In blockage and foliage environments, GNSS faults, such as code outliers, phase slips, and ionosphere disturbances, may occur frequently due to the several multipath and fast-changing environments. The faulty observations violate the assumed GNSS stochastic model and if not properly handled, the integrated solutions would be deteriorated. In the literature, test-based outlier detection and robust estimation are two main streams for dealing with GNSS faults [16,17]. Baarda proposed to apply hypothesis test to detect outliers in geodetic measurement [18]. Teunissen generalized it to recursive detection, identification, and adaptation (DIA) method [19]. The DIA method has found its use in different GPS applications [20,21] and various configurations of integrated navigation systems [19,22]. Unlike DIA, the aim of robust estimation methods is to make the estimate insensitive to outliers or other statistical uncertainties, not to detect/identify/correct the outliers. In robust estimation community, with high efficient and accuracy, M-estimation is the prominent one which receives special attentions [18]. Yang et al. proposed an adaptive robust KF which use M-estimation with fading factor for addressing both the system errors and measurement outliers [23]. Moreover, M-estimation is also employed in nonlinear KF. e.g., the unscented Kalman filter [24]. A robust KF based on the Mahalanobis distances and Chi-square test is devised to detect and then adapt the outliers [25]. This method possesses some features of both the above two kinds and its efficacy was demonstrated in loosely-coupled GNSS/INS integration [26]. However, the method uses all observations to form the test statistics and only one scaling factor is designed to inflate the covariance matrix of the innovation. In TC, it cannot effectively identify which measurement channels contain faults and the high accuracy of good observations will be sacrificed.

With the advent of multi-constellation multi-frequency GNSS, multi-type observations such as pseudorange, Doppler, and carrier phase of multiple satellites are available for measurement update in TC of GNSS/INS integration. The computational burden when processing observation vectors of high dimension must be considered, especially for an embedded CPU. In conventional KF, the observation vector is processed in one single measurement update, which is time consuming due to the inversion of high-dimensional matrix. Alternatively, the high-dimensional observations can be decomposed into multiple low-dimensional or scaler observations and then the measurement update is performed sequentially [27]. The current researches about TC mainly focus on the modeling and robustness enhancement of GNSS/INS integration. Few papers are devoted to analyzing the computation cost of integration algorithms.

Considering the accuracy, robustness, and computational efficiency problems, we propose a robust and efficient implementation of tightly coupled GNSS/INS integration. All type observations, including pseudorange, Doppler, and carrier phase, are fused with INS to achieve the maximum possible navigation accuracy for a single receiver (TC-PDC). To improve the computational efficiency, sequential KF is employed to process the high-dimensional observations. Based on the innovation of sequential KF, a robust estimation method with Gaussian test is designed to detect and adapt the faults in individual channels. The performance of the proposed method, in terms of accuracy, robustness and computational efficiency, is analyzed by two vehicular field tests. Our contribution differs from previous work in the following aspects. (1) A complete performance comparison among LC, TC-PD, and TC-PDC is conducted to show the improvements of position, velocity, and attitude accuracy brought by the TDCP measurement. (2) A sequential robust estimation method is devised to utilize the faulty and good observations rationally. (3) The computation times are analyzed to reveal the superiority of sequential over batch mode.

This paper is organized as follows. Section 2 describes the GNSS observation equations and the error mitigation strategies. Section 3 establishes the mathematical model for TC GNSS/INS integration. The corresponding robust sequential KF is presented in Section 4. The experimental results and analysis are given in Section 5. Finally, Section 6 concludes the whole paper.

## 2. GNSS Observations

The generic basic observation equations for pseudorange, Doppler, and carrier phase are given as follows

(1) $$\begin{array}{l} P_{r}^{s}=\rho_{r}^{s}+c\left(dt_{r}-dt^{s}\right)+I_{r}^{s}+T_{r}^{s}+\varepsilon_{P,r}^{s}\\ \lambda D_{r}^{s}={\dot{\rho }}_{r}^{ij}+c\left(d{\dot{t}}_{r}-d{\dot{t}}^{s}\right)+{\dot{I}}_{r}^{s}+{\dot{T}}_{r}^{s}+\varepsilon_{D,r}^{s}\\ \varphi_{r}^{s}=\rho_{r}^{s}+c\left(dt_{r}-dt^{s}\right)-I_{r}^{s}+T_{r}^{s}+\lambda N_{r}^{s}+\varepsilon_{\Phi ,r}^{s} \end{array}$$

where $P_{r}^{s}$, $D_{r}^{s}$, and $\varphi_{r}^{s}$ are respectively pseudorange, Doppler, and carrier phase observations, $\rho_{r}^{s}$ is the geometric range between a receiver-satellite pair, $dt_{r}$ and $dt^{s}$ are the receiver and satellite clock error, $I_{r}^{s}$ and $T_{r}^{s}$ are the ionospheric and tropospheric delay, $N_{r}^{s}$ is the integer ambiguity, c is the speed of light, λ is carrier phase wavelength, and $\varepsilon_{P,r}^{s}$, $\varepsilon_{D,r}^{s}$, and $\varepsilon_{\Phi ,r}^{s}$ represent the code, Doppler, and carrier phase noise, respectively.

To mitigate the nuisance errors and delays above, difference and combination techniques are adopted. We apply between-satellite single difference to eliminate receiver clock error. For carrier phase, time difference is also added to remove the ambiguity parameter. For pseudorange observation, the ionospheric delay is compensated using the dual-frequency ionosphere-free combination, and the tropospheric delay is mostly corrected by the Saastamoinen model [28]. Considering atmospheric delays are temporally correlated, they less affect the Doppler observable and time difference carrier phase (TDCP). Denoting the between-satellite single difference and time difference operators by

(2) $$\begin{array}{l} {\left(•\right)}^{ij}={\left(•\right)}^{j}-{\left(•\right)}^{i}\\ \Delta_{t}\left(•\right)=\left(•\right)\left(k\right)-\left(•\right)\left(k-1\right) \end{array}$$

then the compensated equations for Equation (1) read

(3) $$\begin{array}{l} P_{r}^{ij}=\rho_{r}^{ij}+cdt^{ij}+{\tilde{I}}_{IF}^{ij}+{\tilde{T}}_{Sa}^{ij}+\varepsilon_{P,r}^{ij}\\ \lambda D_{r}^{ij}={\dot{\rho }}_{r}^{ij}+cd{\dot{t}}^{ij}+\varepsilon_{D,r}^{ij}\\ \Delta_{t}\varphi_{r}^{ij}=\Delta_{t}\rho_{r}^{ij}+c\Delta_{t}dt^{ij}+\Delta_{t}\varepsilon_{\Phi ,r}^{ij} \end{array}$$

with ${\tilde{I}}_{IF}^{ij}$ and ${\tilde{T}}_{Sa}^{ij}$ the calculated ionospheric and tropospheric delays by the above methods.

## 3. Tightly-Coupled GNSS/INS Integration Model

In this paper, GNSS/INS integration is implemented through indirect KF with feedback correction. The linear INS error model augmenting sensor error stochastic process constitutes the system model. The integration KF directly fuses GNSS raw observations and INS-derived counterparts to estimates the error states, which are fed back into the INS to correct the navigation error and sensor biases. In the following, the dynamic model and measurement model for the integration are developed.

### 3.1. Dynamic Model

The INS psi-angle error model reads

(4) $$\{\begin{array}{l} \delta {\dot{r}}^{n}=-\omega_{en}^{n}\times \delta r^{n}+\delta v^{n}\\ \delta {\dot{v}}^{n}=-(2\omega_{ie}^{n}+\omega_{en}^{n})\times \delta v^{n}-\psi \times C_{b}^{n}f^{b}+\delta g^{n}+C_{b}^{n}\nabla^{b}\\ \dot{\psi }=-(\omega_{ie}^{n}+\omega_{en}^{n})\times \psi -C_{b}^{n}\varepsilon^{b} \end{array}$$

where superscripts n and b denote the navigation frame (n-frame) and the body frame (b-frame), respectively; $\delta r^{n}$, $\delta v^{n}$, and ψ are the position, velocity, and attitude error vectors, respectively; $\omega_{ie}^{n}$ is the earth rotation rate with respect to the inertial frame (i-frame); $\omega_{en}^{n}$ is the transport rate representing the turn rate of the n-frame with respect to the earth-centered earth-fixed frame (e-frame); $f^{b}$ is the specific force measured by the accelerometers; $\delta g^{n}$ is the gravity error which can be expressed as the function of the INS position, velocity, and misalignment angle; $C_{b}^{n}$ is the direction cosine matrix (DCM) from b-frame to n-frame; $\nabla^{b}$ and $\varepsilon^{b}$ are the bias errors for accelerometers and gyros, modeled as first-order Gauss–Markov processes

(5) $$\begin{array}{l} {\dot{\nabla }}^{b}=-\frac{1}{T_{a}}\nabla^{b}+\omega_{a}\\ {\dot{\varepsilon }}^{b}=-\frac{1}{T_{g}}\varepsilon^{b}+\omega_{g} \end{array}$$

with $T_{a}$, $T_{g}$ are the respective correlation times, and $\omega_{a}$, $\omega_{g}$ are the respective driven noise for accelerometers and gyros. These parameters can be obtained using Allan variance analysis of inertial sensors [29].

Equations (4) and (5) constitute the dynamic model, which can be written as a compact form

(6) $$\dot{x}=Fx+Gw$$

where F is the dynamic matrix, G is the noise distribution matrix, and x is the state vector, including 9 navigation error states and 6 sensor bias states

(7) $$x={\left[\begin{array}{ccccc} {\left(\delta r^{n}\right)}^{T} & {\left(\delta v^{n}\right)}^{T} & {\left(\psi \right)}^{T} & {\left(\nabla^{b}\right)}^{T} & {\left(\varepsilon^{b}\right)}^{T} \end{array}\right]}^{T}$$

### 3.2. Measurement Model

In order to get the measurement model, the nonlinear observation Equation (3) can be linearized with respect to the unknown receiver positon and velocity

(8) $$\begin{array}{l} P_{OMC}^{ij}=P_{r}^{ij}-\rho_{r,I}^{ij}={\left(e^{ij}\right)}^{T}C_{n}^{e}\delta r_{G}^{n}+\varepsilon_{P,r}^{ij}\\ D_{OMC}^{ij}=D_{r}^{ij}-{\dot{\rho }}_{r,I}^{ij}={\left(e^{ij}\right)}^{T}C_{n}^{e}\delta v_{G}^{n}+\varepsilon_{D,r}^{ij}\\ \Delta_{t}\varphi_{OMC}^{ij}=\Delta_{t}\lambda \varphi_{r}^{ij}-\Delta_{t}\rho_{r,I}^{ij}=e^{ij}{\left(k\right)}^{T}C_{n}^{e}\delta r_{G}^{n}\left(k\right)-e^{ij}{\left(k-1\right)}^{T}C_{n}^{e}\delta r_{G}^{n}\left(k-1\right)+\Delta_{t}\varepsilon_{\Phi ,r}^{ij} \end{array}$$

where $P_{OMC}^{ij}$, $D_{OMC}^{ij}$, and $\Delta_{t}\varphi_{OMC}^{ij}$ denote the observed-minus-computed (OMC) observations, $\rho_{r,I}^{ij}$, ${\dot{\rho }}_{r,I}^{ij}$, and $\Delta_{t}\rho_{r,I}^{ij}$ are the corresponding predicted observations using INS states estimates, $e^{ij}$ is the single-difference line-of-sight vector, $C_{n}^{e}$ is the DCM from n-frame to e-frame, $\delta r_{G}^{n}$ and $\delta v_{G}^{n}$ are the position and velocity error vectors of GNSS antenna phase center.

The position and velocity of GNSS antenna phase center are related to those of IMU center as follows [26]

(9) $$\begin{array}{l} r_{G}^{n}=r^{n}+C_{b}^{n}l^{b}\\ v_{G}^{n}=v^{n}-\left(\omega_{in}^{n}\times \right)C_{b}^{n}l^{b}-C_{b}^{n}\left(l^{b}\times \right)\omega_{ib}^{b} \end{array}$$

where $r_{G}^{n}$ and $v_{G}^{n}$ represent the position and velocity of GNSS antenna phase center, $r^{n}$ and $v^{n}$ are the position and velocity of IMU center, $\omega_{in}^{n}=\omega_{ie}^{n}+\omega_{en}^{n}$ is the rotation rate of n-frame with respect to the i-frame, $\omega_{ib}^{b}$ is the angular rate of b-frame with respect to i-frame measured by the gyros, $l^{b}$ is the lever arm, and (·×) is the skew-symmetric matrix. The corresponding position and velocity error vectors of GNSS and INS can be further derived as

(10) $$\begin{array}{l} \delta r_{G}^{n}=\delta r^{n}+\left(C_{b}^{n}l^{b}\times \right)\psi \\ \delta v_{G}^{n}=\delta v^{n}-\left[\left(\omega_{in}^{n}\times \right)C_{b}^{n}\left(l^{b}\times \right)+C_{b}^{n}(l^{b}\times \omega_{ib}^{b})\times \right]\psi -C_{b}^{n}\left(l^{b}\times \right)\varepsilon^{b} \end{array}$$

Defining the following matrices

(11) $$\begin{array}{l} A_{r}=\left[\begin{array}{ccccc} I_{3} & 0_{3\times 3} & C_{b}^{n}l^{b}\times & 0_{3\times 3} & 0_{3\times 3} \end{array}\right]\\ A_{v}=\left[\begin{array}{ccccc} 0_{3\times 3} & I_{3} & -\left[\left(\omega_{in}^{n}\times \right)C_{b}^{n}\left(l^{b}\times \right)+C_{b}^{n}(l^{b}\times \omega_{ib}^{b})\times \right] & 0_{3\times 3} & -C_{b}^{n}\left(l^{b}\times \right) \end{array}\right] \end{array}$$

and substituting Equations (10) and (11) into Equation (8) yields

(12) $$\begin{array}{l} P_{OMC}^{ij}={\left(e^{ij}\right)}^{T}C_{n}^{e}A_{r}x+\varepsilon_{P,r}^{ij}\\ D_{OMC}^{ij}={\left(e^{ij}\right)}^{T}C_{n}^{e}A_{v}x+\varepsilon_{D,r}^{ij} \end{array}$$

The carrier phase equation in Equation (8) is related to both the current and past position errors, whichviolates the assumption of a normal KF. In this deduction, we perform backward time propagation to reformulate the past state as

(13) $$x\left(k-1\right)=\Phi_{k,k-1}^{-1}x\left(k\right)$$

where $\Phi_{k,k-1}$ is the state transition matrix from epoch *k* − 1 to *k*. Using Equation (13), the linearized phase equation can be expressed as

(14) $$\Delta_{t}\varphi_{OMC}^{ij}=\left(e^{ij}{\left(k\right)}^{T}C_{n}^{e}A_{r}-e^{ij}{\left(k-1\right)}^{T}C_{n}^{e}A_{r}\Phi_{k,k-1}^{-1}\right)x+\Delta_{t}\varepsilon_{\Phi ,r}^{ij}$$

Based on Equations (12) and (14), the measurement model for tightly-coupled GNSS/INS integration is given as

(15) Z=Hx+ε

where

(16) $$Z=\left[\begin{array}{c} P_{OMC}^{ij}\\ D_{OMC}^{ij}\\ \Delta_{t}\varphi_{OMC}^{ij} \end{array}\right]$$

H is the measurement matrix given by

(17) $$H=\left[\begin{array}{c} {\left(e^{ij}\right)}^{T}C_{n}^{e}A_{r}\\ {\left(e^{ij}\right)}^{T}C_{n}^{e}A_{v}\\ e^{ij}{\left(k\right)}^{T}C_{n}^{e}A_{r}-e^{ij}{\left(k-1\right)}^{T}C_{n}^{e}A_{r}\Phi_{k,k-1}^{-1} \end{array}\right]$$

and $\varepsilon =\left[\begin{array}{c} \varepsilon_{P,r}^{ij}\\ \varepsilon_{D,r}^{ij}\\ \Delta_{t}\varepsilon_{\Phi ,r}^{ij} \end{array}\right]$ is the observation noise vector.

## 4. Robust Sequential KF

With the development of multi-constellation multi-frequency GNSS, at each measurement update epoch, an increasing number of pseudorange, Doppler, and carrier phase observations are available. When implementing tightly coupled GNSS/INS integration, the computational efficiency needs to be considered. What is also important is that prior to processing any type of observation, a health check should be performed to detection and handle faults.

### 4.1. Sequential KF

For the Kalman filtering processing of the GNSS observations, two options exist. One is batch processing. All the observations form a vector which is then processed in one single measurement update. The other option is sequential processing which performs a scalar measurement update for each observation. In batch processing, a matrix with the number of rows equal to the number of available observations has to be inverted in the calculation of the Kalman gain matrix. Whereas in sequential processing, the matrix inversion reduces to a simple division, which not only improves the computational efficiency but also guarantees the stability of numerical calculation in the case of massive observations. Moreover, the sequential processing enables detection of faults in individual GNSS channels, which will be shown in the next subsection.

The time update step is the same for both sequential mode and batch processing

(18) $$\begin{array}{l} {\hat{x}}_{k/k-1}=\Phi_{k,k-1}{\hat{x}}_{k-1}\\ P_{k/k-1}=\Phi_{k,k-1}P_{k-1}\Phi_{k,k-1}^{T}+Q_{k-1} \end{array}$$

where ${\hat{x}}_{k-1}$ and $P_{k-1}$ are the estimated state vector and the corresponding covariance matrix, $Q_{k-1}$ stands for the process noise covariance matrix. Assuming that totally m observations are available at epoch *k*, let

(19) $$\begin{array}{l} Z_{k}={\left[\begin{array}{cccc} z_{k}^{1} & z_{k}^{2} & \cdots & z_{k}^{m} \end{array}\right]}^{T}\\ H_{k}={\left[\begin{array}{cccc} h_{k}^{1}{}^{T} & h_{k}^{2}{}^{T} & \cdots & h_{k}^{m}{}^{T} \end{array}\right]}^{T}\\ R_{k}=diag\left(\left[\begin{array}{cccc} r_{k}^{1} & r_{k}^{2} & \cdots & r_{k}^{m} \end{array}\right]\right) \end{array}$$

The measurement update steps then proceed with each observation sequentially

(20) $$\begin{array}{cc} \begin{array}{l} k_{k}^{i}=P_{k}^{i-1}h_{k}^{i}{}^{T}{\left(h_{k}^{i}P_{k}^{i-1}h_{k}^{i}{}^{T}+r_{k}^{i}\right)}^{-1}\\ {\hat{x}}_{k}^{i}={\hat{x}}_{k}^{i-1}+k_{k}^{i}\left(z_{k}^{i}-h_{k}^{i}{\hat{x}}_{k}^{i-1}\right)\\ P_{k}^{i}=\left(I-k_{k}^{i}h_{k}^{i}\right)P_{k}^{i-1} \end{array} & i=1,2,\cdots ,m \end{array}$$

with the initial state and its covariance matrix given by

(21) $$\begin{array}{l} {\hat{x}}_{k}^{0}={\hat{x}}_{k/k-1}\\ P_{k}^{0}=P_{k/k-1} \end{array}$$

### 4.2. Robust Estimation Based on Innovation Test

It is well-known that the KF innovation is Gaussian distributed with zero mean. The GNSS faults would violate this property and thus degenerate the filter. In the worst case, the filter may diverge. In GNSS/INS integration, it is very important to resist this adverse influence of GNSS faults.

According to Equation (20), in the *i*-th measurement update at epoch k, The KF innovation $v_{k}^{i}$ and the corresponding variance $\sigma_{i}^{2}$ are

(22) $$\begin{array}{l} v_{k}^{i}=Z_{k}^{i}-h_{k}^{i}{\hat{x}}_{k}^{i-1}\\ \sigma_{i}^{2}=h_{k}^{i}P_{k}^{i-1}h_{k}^{i}{}^{T}+r_{k}^{i} \end{array}$$

where ${\hat{x}}_{k}^{i-1}$ is the estimated state after the (i−1)th measurement update. In the absence of faults, the mean of innovation will be zero. Otherwise, the mean will be shifted. Therefore, the hypothesis test can be conducted to detect the faults with null hypothesis $H_{0}$ and alternative hypothesis $H_{1}$ chosen as

(23) $$\begin{array}{l} H_{0}:v_{k}^{i}∼N\left(0,\sigma_{i}^{2}\right)\\ H_{1}:v_{k}^{i}∼N\left(\delta ,\sigma_{i}^{2}\right) \end{array}$$

where δ is the fault. The test statistic is defined as

(24) $$t=\frac{v_{k}^{i}}{\sigma_{i}}$$

t obeys standard normal distribution and Equation (23) changes to

(25) $$\begin{array}{l} H_{0}:t∼N\left(0,1\right)\\ H_{1}:t∼N\left(\delta^{\prime },1\right) \end{array}\;\mathrm{with}\;\delta^{\prime }=\frac{\delta }{\sigma_{i}}.$$

The threshold can be determined using the distribution probability function to meet the required probability of false alert. A fault will be detected if the test statistic and the threshold meet

(26) $$\left|t\right|>T_{d}$$

**Figure 1:**
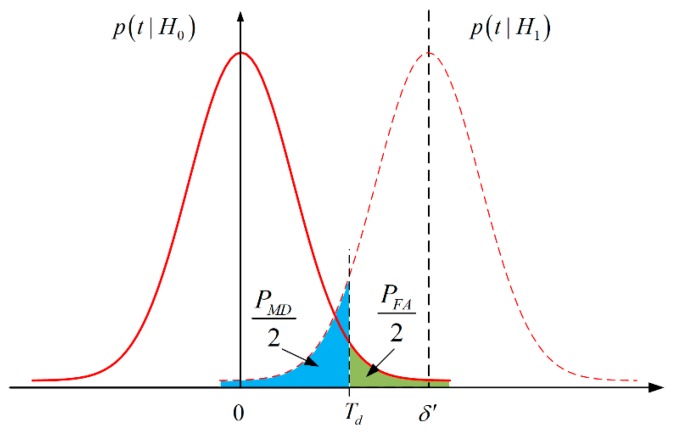
Probability density functions of hypotheses test for fault detection.

For a given probability of false alert α, the threshold $T_{d}$ can be given by the $1-\frac{\alpha }{2}$ quantile $n_{1-\alpha /2}$ of standard normal distribution function, i.e., $T_{d}=n_{1-\alpha /2}$, which means that the decision is made at the risk of α. As shown in Figure 1, the test threshold controls the probabilities of false alert and miss detection, and there is a tradeoff between them. An increase in the threshold implies a lower false alarm rate and higher miss detection rate, and vice versa. Generally, the required probability of false alert is chosen by rule of thumb. In this paper, the value of α is set as 0.001.

Once a fault is detected, then the following scaling factor is calculated to inflate the variance

(27) $$\begin{array}{l} \kappa =\frac{\left|t\right|}{n_{1-\alpha /2}}\\ \kappa^{2}\sigma_{i}^{2}\to \sigma_{i}^{2} \end{array}$$

$\kappa^{2}$ is the scaling factor which indicates the discrepancy between the actual variance and its theoretical value. By inflating the variance, the detected faulty measurement can be downweighted in the update process of KF. The KF gain in Equation (20) can then be adaptively adjusted to resist the adverse effects of GNSS faults. After processing the previous (i−2)th observations, ${\hat{x}}_{k}^{i-1}$ is more accurate than ${\hat{x}}_{k}^{i-2}$, and of course more accurate than the predicted value ${\hat{x}}_{k/k-1}$, i.e., $P_{k}^{i-1}\leq P_{k}^{i-2}\cdots \leq P_{k/k-1}$, leading to a larger $\delta^{\prime }$ of Equation (25). As shown in Figure 1, a larger $\delta^{\prime }$ makes less intersect areas, so smaller miss detection probability $P_{MD}$ could be achieved. Consequently, it is more probable to correctly detect the fault. Hence, better reference information (more accurate ${\hat{x}}_{k}^{i}$) implies a better capability of detecting faults. However, in robust KF with batch processing [26], only the predicted ${\hat{x}}_{k/k-1}$ and $P_{k/k-1}$ are available. It can then be concluded that robust sequential KF could not only detect faults in individual channels, but also possess better detection capability.

**Figure 2:**
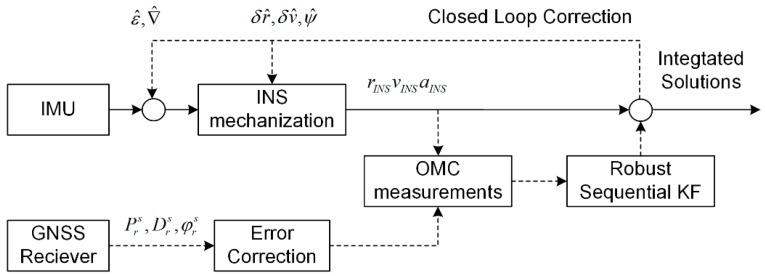
Tightly coupled Global Navigation Satellite System (GNSS)/Inertial Navigation System (INS) integration through a robust sequential Kalman Filter (KF).

Figure 2 shows the block diagram of the proposed tightly-coupled GNSS/INS integration algorithm. In the figure, the solid and dash arrows are the INS-related high-rate and GNSS-related low-rate information flows, respectively. INS mechanization calculates the high-rate navigation result (position, velocity, and attitude) with the compensated raw INS data. In parallel, the GNSS receiver provides low-rate pseudorange, Doppler, carrier phase, and ephemeris data. Then systematic error sources are corrected using combination and difference techniques, along with some empirical models. The corrected GNSS observations and the INS-predicted counterparts form the OMC measurements. The robust sequential KF uses the OMC measurements to estimate the error states, which are fed back into the INS to correct the navigation solution as well as the INS sensor biases.

## 5. Test and Results

In this section, two vehicular test results are presented. The first one compares the performance of different integration and update processing strategies in terms of accuracy and efficiency in favorable environments. The second test focuses on testing the robustness of the algorithm in harsh environments. For both tests, MEMS- and high-grade IMUs were used. The data from MEMS-grade IMU is processed using the proposed algorithm. The data collected by the base station and high-grade IMU is post processed using RTK/INS integration with backward smoothing by the GINS software of Wuhan MP Space Time Technology Company, which is used as a reference. For better observability of the system state, the vehicle run along trajectories on which there are numerous motion types (acceleration, deceleration, and turn).

### 5.1. Test #1

**Figure 3:**
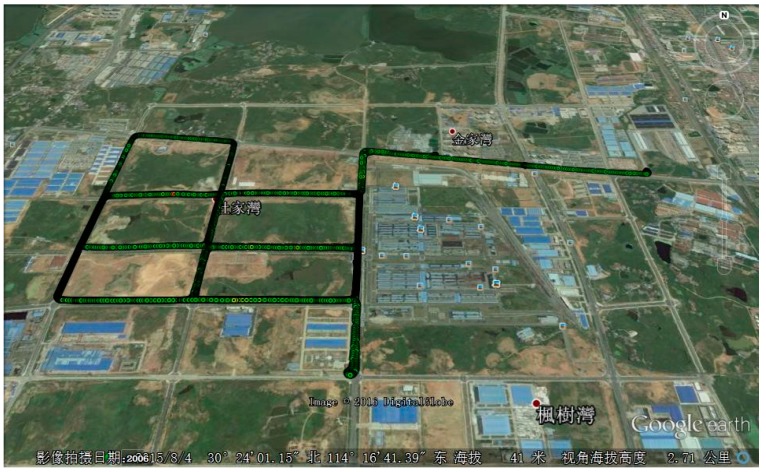
Trajectory of test #1.

**Figure 4:**
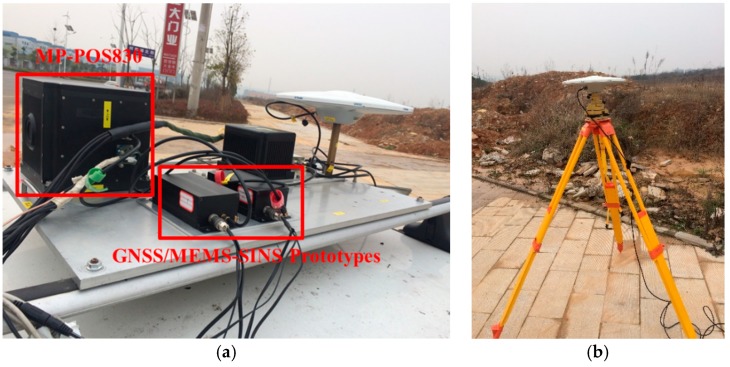
Test setup. (**a**) Equipment mounted on the roof of a car; (**b**) Base station.

The test was performed in March 2015 in a suburb of Wuhan, China, as shown in Figure 3. Two sets of navigation systems were mounted on the roof of the test vehicle. The first is a prototype GNSS/INS system which integrates low-cost STIM300 MEMS with Novatel OEMV-3 receiver. The raw data from the prototype system was processed and analyzed with the proposed algorithm. The sampling rates were set 125 Hz and 1 Hz for MEMS and GNSS receiver, respectively. The second is a reference system (POS830, manufactured by Wuhan MP Space Time Technology Company, Wuhan, China). A temporary base station was set up nearby whose coordinate was determined through PPP processing of 2-h static GPS/GLONASS/BDS data. The resultant solutions act as the reference to evaluate the performance of the proposed algorithm when applied to MEMS-based INS. Figure 4 shows the test setup. Table 1 and Table 2 list the performance parameters of the two systems.

#### 5.1.1. Satellite Availability

**Figure 5:**
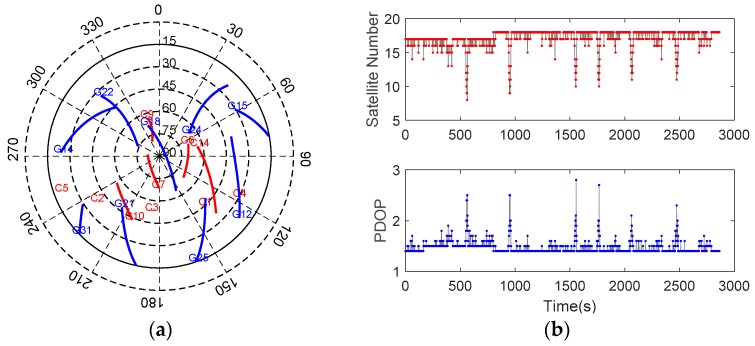
Sky plot (**a**) and the satellite visibility (**b**) for test #1.

The GNSS receiver was set to track GPS (L1 and L2) and BDS (B1, B2, and B3) dual-constellation five-frequency signals. Figure 5 shows the sky plot and the satellite visibility in terms of satellite number and positional dilution of precision (PDOP) (15° elevation mask). It can be observed that the geometry is favorable, and the average number of visible satellites and PDOP are 17.26 and 1.46, respectively. This indicates that multi-GNSS combinations can raise the number of available satellites and strength the station-satellite geometry significantly.

To clearly show the advantages of the proposed algorithm, we processed the data in three integration schemes: loose coupling (LC), tight coupling based on pseudorange and Doppler (TC-PD), and tight coupling based on pseudorange, Doppler, and carrier phase (TC-PDC). In addition, batch and sequential measurement update strategies were all implemented. We first compare the accuracy performance of the three schemes. The enhancement of the robust estimation in the presence of GNSS faults, especially during complete GNSS outages, is analyzed in the sequel. Finally, the computational speed of the two measurement update strategies is compared to reveal the improvement of computational efficiency brought by the sequential KF.

#### 5.1.2. Performance Comparison: LC vs. TC-PD vs. TC-PDC

**Figure 6:**
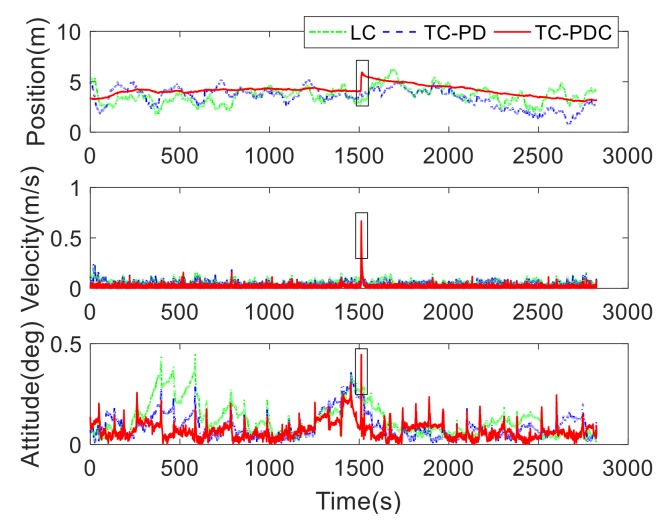
Navigation errors of loose coupling (LC), tight coupling based on pseudorange and Doppler (TC-PD), and tight couple based on pseudorange, Doppler, and carrier phase (TC-PDC).

Figure 6 plots the position, velocity and attitude errors of the three integration schemes. Table 3 summarizes the corresponding root-mean-square errors (RMSE). Compared with LC, the RMSE values of TC-PD decrease from 3.7490 m, 0.0601 m/s, and 0.1463 deg to 3.5867 m, 0.0538 m/s, and 0.1086 deg with improvements of 4.33%, 10.48%, and 25.77% in the position, velocity, and attitude. As expected, TC outperforms LC. For TC-PDC, the RMSE values of velocity and attitude further decrease to 0.0272 m/s and 0.0795 deg, with significant improvements of 54.74% and 45.66%. However, the position accuracy becomes a bit worse with a degradation of 10.93%. From Figure 6, the position errors of TC-PDC abruptly jumps around 1555 s (marked with a black rectangle), making the succeeding position errors increase. The velocity and attitude are also observed gross errors around the same time instant. This abnormal errors could be caused by the cycle slip of carrier phase, as they are absent for LC and TC-PD. These outlying navigation results will cause unacceptable degradations in the control performance, especially for some high-speed safety-related applications, thus have to be carefully addressed.

#### 5.1.3. Performance of Robust Estimation

**Figure 7:**
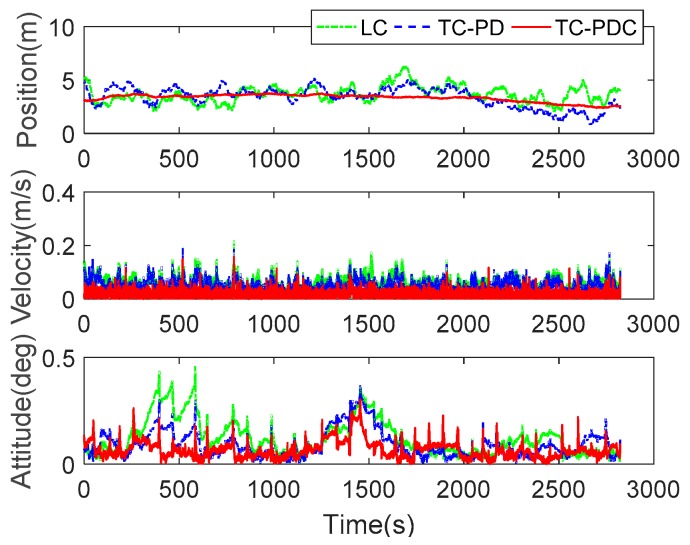
Navigation errors of LC, TC-PD, and TC-PDC with robust KF.

For the same data set, robust estimation is applied to resist the faults in GNSS observations. Figure 7 illustrates the performance of robust KF for the three integration schemes, and Table 4 summarizes the corresponding RMSE. Comparing Figure 6 with Figure 7, the jump errors of TC-PDC disappear. The proposed robust estimation method eliminates the abnormal navigation errors. The position error of TC-PDC is more stable than the two counterparts, which leads to better accuracy of position increments. Comparing Table 3 with Table 4, with robust KF, the position and velocity accuracy of TC-PDC are significantly improved by 19.65% (from 4.1588 m to 3.3416 m) and 34.56% (from 0.0272 m/s to 0.0178 m/s); For TC-PD, the robust KF slightly improves the position and velocity accuracy by 1.42% (from 3.5867 m to 3.5359 m) and 6.13%(from 0.0538 m/s to 0.0505 m/s); For LC, only velocity accuracy achieves a slight improvement of 3.16% (from 0.0611 m/s to 0.0593 m/s). These results demonstrate the superiority of robust estimation in detecting and resisting the faults. For the three integration schemes with robust KF, the RMSE of LC decrease from 3.7526 m, 0.0582 m/s, and 0.1478 deg to 3.3416 m, 0.0178 m/s, and 0.0781 deg of TC-PDC with improvements of 10.95%, 69.42% and 47.16%. Additionally, robust TC-PDC is also superior to robust TC-PD, which improves the navigation accuracy by 5.50%, 64.75% and 30.88% for position, velocity and attitude, respectively. Obviously, the introduction of time-differenced carrier phase remarkably improved the velocity accuracy. The overall attitude accuracy also benefits.

**Figure 8:**
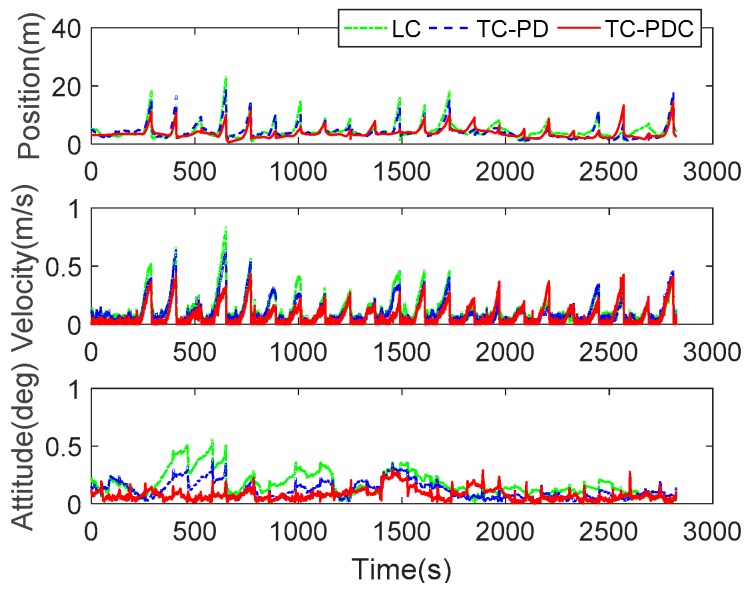
Navigation errors of LC, TC-PD, and TC-PDC with several 60-s simulated GNSS outages.

According to Figure 5, the preceding results are obtained in a favorable observation environment (The minimal number of visible satellites are greater than 5). To assess the performance of the proposed algorithm in GNSS denied and degraded areas (urban canyons or open pit mines), several complete GNSS outages, each lasting 60 s, are simulated after the navigation filter converges. The simulated outages are implemented by masking all the visible satellites artificially. During these outages, the navigation solution is solely dependent on inertial navigation, i.e., no closed-loop correction during the 60 s. Figure 8 compares the navigation errors among the three integration schemes. TC-PDC achieves smallest navigation errors in most cases, followed by TC-PD and LC. The corresponding RMSE and maximum errors are listed in Table 5. The statistics indicate that the maximum navigation errors of LC decrease from 23.3554 m, 0.8399 m/s, and 0.5647 deg to 18.6719 m, 0.6682 m/s, and 0.3851 deg of TC-PD with improvements of 20.05%, 20.44%, and 31.80%, and further decease to 14.1924 m, 0.4416 m/s, and 0.3071 deg of TC-PDC with improvements of 39.23%, 47.42%, and 45.62%. The results show that TC-PDC possess better stand-alone INS positioning capability, which benefits from the superior velocity and attitude accuracy of time-differenced carrier phase aiding.

#### 5.1.4. Computational Efficiency Comparison: Sequential vs. Batch

The above results demonstrate that TC-PDC outperforms LC and TC-PD in terms of navigation performance. For real-time implementation of integration algorithm, an important concern is the computational cost. It is necessary to compare the computation times of different processing strategies for the measurement update of the three integration schemes. For comparison purpose, all the measurements are processed in a normal way, i.e., no fault detection and adaption, which corresponds to Section 5.1.2. All the algorithms were developed in ANSI C language and all computations were performed on an Intel Core i5 3.30 GHz PC with 8 GB memory running Windows 7.

**Figure 9:**
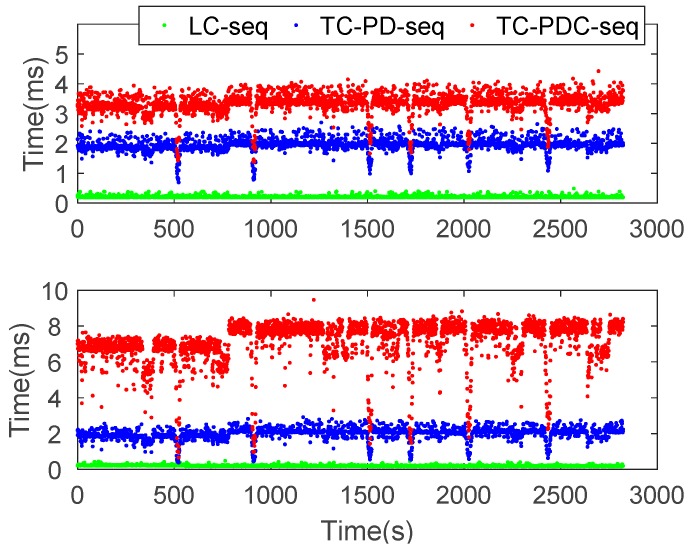
Computation times of measurement update for LC, TC-PD, and TC-PDC.

Figure 9 compares the computation times of measurement update for the three integration schemes. The top and bottom panels correspond to the computation times of sequential and batch modes, respectively. As can be seen in the figure, TC-PDC is the slowest, followed by TC-PD. LC has extremely low computational times (lower than 1 ms). The sequential mode impressively improves the computational efficiency for TC-PDC, whereas this is not the case for LC and TC-PD. Table 6 reports the mean value of computation times. Compared with batch processing, the sequential processing significantly improves the computational efficiency for TC-PDC by 53.10% (from 7.098 ms to 3.329 ms). For TC-PD, there is a slight improvement, just 1.87% (from 1.979 ms to 1.942 ms). However, the mean computation time for LC adversely increases a bit.

**Figure 10:**
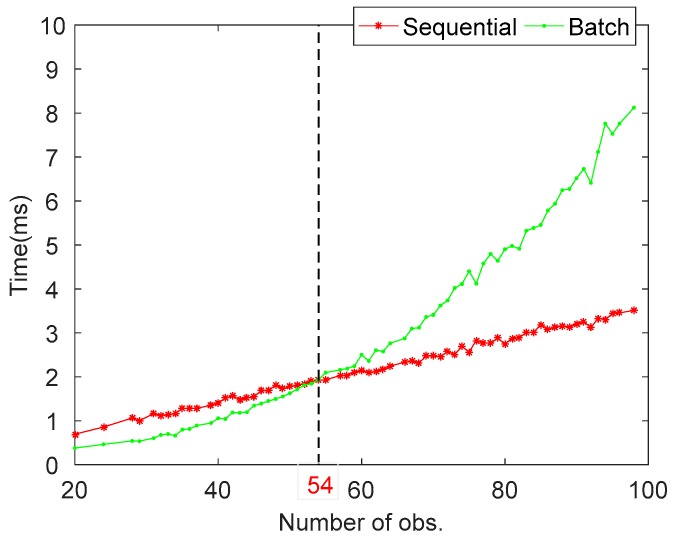
Computation times versus number of observations for batch and sequential modes.

Figure 10 plots the mean computation times with the increase of number of observations in TC-PD and TC-PDC for sequential and batch processing. As the number of observations increases, the computation times of batch and sequential modes increase in polynomial and linear ways, respectively. When the number of observations are more than about 54, the sequential mode is faster than batch mode and this superiority is more significant for a large number of observations. The main reason behind is that the computational cost of matrix inversion in batch mode proportional to the third order of dimension. From Equation (20), it is easy to conclude that the computation times of measurement update in sequential mode proportional to the number of observations to be processed. Therefore, it is suggested that for multi-constellation multi-frequency GNSS/INS tightly coupled integration, sequential measurement update should be adopted to improve the computational efficiency.

### 5.2. Test #2

**Figure 11:**
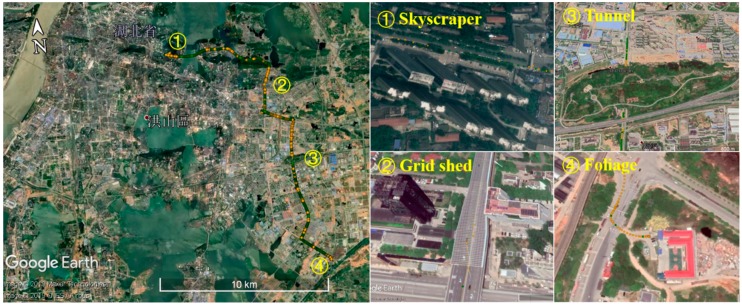
Trajectory and four typical GNSS challenging environments of test #2.

The second test was performed in a city urban environment in Wuhan, China, on 11 September 2018, as shown in Figure 11. The trajectory was generated using RTKLIB program package (http://www.rtklib.com), where green and yellow dots stand for fixed and float solutions, respectively. The vehicle drove four typical GNSS changeling environments successively, including

① Skyscraper: 18 s–63 s
② Grid shed: 573 s–647 s
③ Tunnel: 1139 s–1186 s
④ Foliage: 2142 s–2225 s

**Figure 12:**
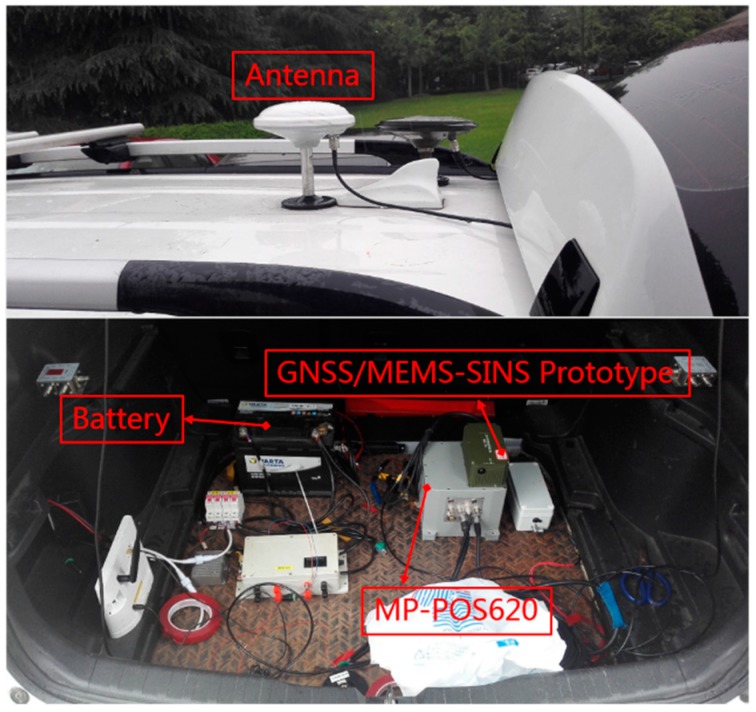
Equipment for test #2.

The test equipment is shown in Figure 12. The GNSS/MEMS-SINS Prototype consists of STIM 300 MEMS and ComNav K708 receiver. The sampling rates of INS measurements and GNSS observations were 125 Hz and 10 Hz, respectively. The collected data was processed and analyzed in the sequel. POS620 from Wuhan MP Space Time Technology Company was used for generating the reference. The corresponding main performance parameters of POS620 are shown in Table 7.

#### 5.2.1. Satellite Availability

**Figure 13:**
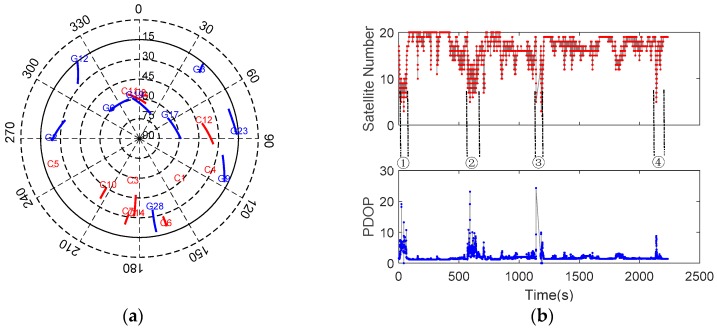
Sky plot (**a**) and the satellite visibility (**b**) for test #2.

Figure 13 shows the sky plot distribution of available dual-constellation (GPS + BDS) satellites and the corresponding satellite number and PDOP during the test. Compared with test #1, the satellite number varies frequently and the geometry is poor (less visible satellites and large PDOP value) during the four typical environments. The minimal satellite number is less than 4. Especially in the third environment (Tunnel), a complete satellite outage is observed (about 36 s).

As is well known, LC is inferior to TC in GNSS challenging areas where the available satellites is less than 4. Test #1 also demonstrates the superiority of TC over LC. Therefore, we only compared the results of two TC integration schemes: TC-PD and TC-PDC and it reveals the importance of robust estimation in TC, especially in GNSS challenging environments.

#### 5.2.2. Performance in GNSS Challenging Environments

**Figure 14:**
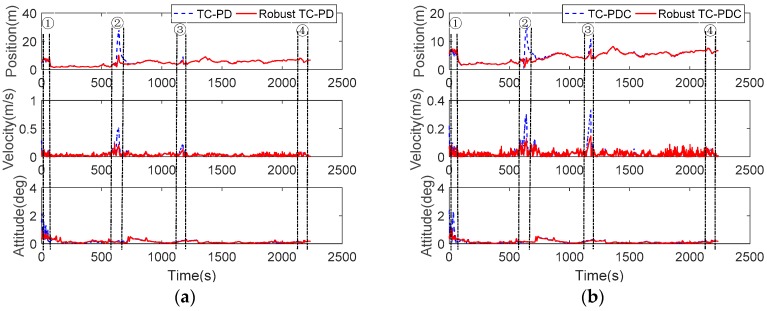
Performance of the proposed method in challenging environments. (**a**) TC-PD and robust TC-PD; (**b**) TC-PDC and robust TC-PDC.

For comparison, the TC solutions without robust estimation were also calculated as plotted in Figure 14. The corresponding RMSE and maximum errors are listed in Table 8. As expected, robust estimation significantly improves the accuracy of TC-PD and TC-PDC, especially in GNSS challenging environments (gird shed and tunnel). Take the position as example, the maximum errors decrease from 27.7496 m to 10.0729 m for TC-PD, and 15.4275 m to 8.1609 m for TC-PDC. Slight improvements are achieved in the foliage scenario as the observation environments are more moderate, as indicated by the satellite number and PDOP. Concerning the error statistics, the accuracy improvements obtained from robust estimation are 11.19%, 34.89%, and 14.60% for TC-PD, and 5.67%, 37.72%, and 29.04% for TC-PDC, respectively, in the position, velocity, and attitude. In addition, it can be observed that robust TC-PDC is superior to robust TC-PD rival, which improves the navigation accuracy by 3.49% for position, 29.53% for velocity, and 1.47% for attitude. The results demonstrate that the robust estimation can significantly promote the TC integration accuracy in GNSS challenging areas, especially for the velocity and attitude components.

## 6. Conclusions

With the rise of multi-constellation multi-frequency GNSS, tightly coupled integration of GNSS/INS benefits a lot in terms of accuracy, availability, and reliability. However, some nuisance issues also arise such as high computation burden and frequently encountered GNSS observation faults. In this research, we proposed a tightly coupled GNSS/INS integration algorithm using robust sequential KF for accurate vehicular navigation. Apart from pseudorange and Doppler used in traditional tight coupling, the time differenced carrier phase measurement is also incorporated to implement GNSS/INS data fusion. Considering the high dimension of observations, the measurement update step in KF is performed in a sequential way to improve the computational efficiency. Based on this sequential KF innovation, a robust estimation method is further developed to detect and resist the faults in individual channels. By doing so, the good observations can be kept as much as possible. Test results of suburban and urban field experiments are presented and performance comparison with traditional LC and TC-PD is made to validate the effectiveness and superiority of the proposed algorithm. Based on the results and analysis above, we can draw the following conclusions:

(1) Compared with TC-PD and LC, TC-PDC can significantly improves the velocity and attitude accuracy of integrated solutions, and further smooths the positioning error, which shows the superiority of the introduction of high-precision time differenced carrier phase observations.
(2) The innovation-based robust estimation method could effectively detect and resist the GNSS faults in individual channels, which guarantees the reliability of state estimation in GNSS challenging environments. With high-precision time-differenced carrier phase and robust estimation method, TC-PDC also shows better navigation accuracy even during long GNSS outages.
(3) For sequential KF, the computation cost will increase linearly with the increasing of the number of observations, while for batch KF, it is approximately proportional to the third order of the number of observations. The computational efficiency can be improved to a great extent if there are a large number of observations. Therefore, when implementing multi-constellation multi-frequency multi-type-observation tightly couple GNSS/INS integration, it is advised to adopt sequential KF to improve the computational efficiency.

In the future, the algorithm will be implemented on an embedded system consisting of low-cost MEMS and receiver to provide real-time navigation data, which plays a key role in vehicular applications. In addition, the integration with multiple auxiliary sensors such as odometer, radars, digital maps, cameras, etc., will also be considered as future work.

## Figures and Tables

**Table 1:** STIM-300 Micro-Electro-Mechanical Systems (MEMS) specifications.

	Gyroscope	Accelerometer
Bias	250.0 deg/h	0.75 mg
Bias Instability ^a^	0.5 deg/h	0.05 mg
Random Walk ^a^	0.15 deg/√h	0.06 m/s/√h
Linearity	25 ppm	100 ppm

^a^ These parameters are obtained by Allan variance analysis.

**Table 2:** POS830 reference system performance parameters.

Post-processed Accuracy	Position	≤0.05 m
	Velocity	0.02 m/s
	Heading	0.005 deg
	Leveling	0.003 deg
Gyroscope	Bias	0.01 deg/h
	Bias stability	0.01 deg/h
Accelerometer	Bias	25 mGal
	Bias stability	10 mGal

**Table 3:** Root-mean-square errors (RMSE) comparison among LC, TC-PD, and TC-PDC.

	Position (m)	Velocity (m/s)	Attitude (deg)
LC	3.7490	0.0601	0.1463
TC-PD	3.5867	0.0538	0.1086
TC-PDC	4.1588	0.0272	0.0795

**Table 4:** RMSE comparison among LC, TC-PD, and TC-PDC with robust KF.

	Position (m)	Velocity (m/s)	Attitude (deg)
LC	3.7526	0.0582	0.1478
TC-PD	3.5359	0.0505	0.1130
TC-PDC	3.3416	0.0178	0.0781

**Table 5:** RMSE and maximum errors of LC, TC-PD, and TC-PDC with 60-s simulated GNSS outages.

	Position (m)		Velocity (m/s)		Attitude (deg)	
	RMSE	Max	RMSE	Max	RMSE	Max
LC	5.2718	23.3554	0.1643	0.8399	0.2024	0.5647
TC-PD	4.7933	18.6719	0.1440	0.6682	0.1373	0.3851
TC-PDC	3.9664	14.1924	0.1076	0.4416	0.0923	0.3071

**Table 6:** Mean value of computation times for LC, TC-PD, and TC-PDC.

	Batch (ms)	Sequential (ms)
LC	0.201	0.212
TC-PD	1.979	1.942
TC-PDC	7.098	3.329

**Table 7:** POS620 reference system performance parameters.

Post-processed Accuracy	Position	≤0.05 m
	Velocity	0.02 m/s
	Heading	0.012 deg
	Leveling	0.008 deg
Gyroscope	Bias	≤0.02 deg/h
	Bias stability	≤0.02 deg/h
Accelerometer	Bias	≤0.01 mg
	Bias stability	≤0.01 mg
Random Walk	0.003 deg/√h	0.03 m/s/√h

**Table 8:** RMSE and maximum errors in GNSS challenging environments.

	Position (m)		Velocity (m/s)		Attitude (deg)	
	RMSE	Max	RMSE	Max	RMSE	Max
TC-PD	5.7430	27.7496	0.0619	0.5154	0.2076	2.1695
Robust TC-PD	5.0714	10.0729	0.0403	0.2340	0.1773	0.9166
TC-PDC	5.1887	15.4275	0.0456	0.3331	0.2462	2.5374
Robust TC-PDC	4.8944	8.1609	0.0284	0.1478	0.1747	1.1152

## References

[1] Vagle N., Broumandan A., Lachapelle G. (2018). Multiantenna GNSS and Inertial Sensors/Odometer Coupling for Robust Vehicular Navigation. IEEE Internet Things J..

[2] Zhao S., Chen Y., Farrell J.A. (2016). High-Precision Vehicle Navigation in Urban Environments Using an MEM’s IMU and Single-Frequency GPS Receiver. IEEE Trans. Intell. Transp. Syst..

[3] Groves P.D. (2013). Principles of GNSS, Inertial, and Multisensor Integrated Navigation Systems.

[4] Falco G., Pini M., Marucco G. (2017). Loose and Tight GNSS/INS Integrations: Comparison of Performance Assessed in Real Urban Scenarios. Sensors.

[5] Teunissen P.J.G., Montenbruck O. (2017). Springer Handbook of Global Navigation Satellite Systems.

[6] Han H., Wang J. (2017). Robust GPS/BDS/INS tightly coupled integration with atmospheric constraints for long-range kinematic positioning. GPS Solut..

[7] Li T., Zhang H., Niu X., Gao Z. (2017). Tightly-Coupled Integration of Multi-GNSS Single-Frequency RTK and MEMS-IMU for Enhanced Positioning Performance. Sensors.

[8] Han H., Xu T., Jian W. (2016). Tightly Coupled Integration of GPS Ambiguity Fixed Precise Point Positioning and MEMS-INS through a Troposphere-Constrained Adaptive Kalman Filter. Sensors.

[9] Gao Z., Ge M., Shen W., Li Y., Chen Q., Zhang H., Niu X. (2017). Evaluation on the impact of IMU grades on BDS + GPS PPP/INS tightly coupled integration. Adv. Space Res..

[10] Liu S., Sun F., Zhang L., Li W., Zhu X. (2016). Tight integration of ambiguity-fixed PPP and INS: Model description and initial results. GPS Solut..

[11] Li Z., Gao J., Wang J., Yao Y. (2017). PPP/INS tightly coupled navigation using adaptive federated filter. GPS Solut..

[12] Wendel J., Trommer G.F. (2004). Tightly coupled GPS/INS integration for missile applications. Aerosp. Sci. Technol..

[13] Soon B.K.H., Scheding S., Lee H.K., Lee H.K., Durrant-Whyte H. (2008). An approach to aid INS using time-differenced GPS carrier phase (TDCP) measurements. GPS Solut..

[14] Han S., Wang J. (2012). Integrated GPS/INS navigation system with dual-rate Kalman Filter. GPS Solut..

[15] Zhao Y. (2017). Applying Time-Differenced Carrier Phase in Non-Differential GPS/IMU Tightly-Coupled Navigation Systems to Improve the Positioning Performance. IEEE Trans. Veh. Technol..

[16] Knight N.L., Wang J. (2009). A Comparison of Outlier Detection Procedures and Robust Estimation Methods in GPS Positioning. J. Navig..

[17] Yu H., Shen Y., Yang L., Nie Y., Yu H., Shen Y., Yang L., Nie Y. (2019). Robust M-estimation using the equivalent weights constructed by removing the influence of an outlier on the residuals. Surv. Rev..

[18] Huber P.J. (1964). Robust estimation of a location parameter. Ann. Math. Stat..

[19] Teunissen P.J.G. (1990). Quality Control in Integrated Navigation Systems. IEEE Aerosp. Electron. Syst. Mag..

[20] Hewitson S., Wang J. (2006). GNSS receiver autonomous integrity monitoring (RAIM) performance analysis. GPS Solut..

[21] Drevelle V., Bonnifait P. (2011). A set-membership approach for high integrity height- aided satellite positioning. GPS Solut..

[22] Hewitson S., Wang J. (2010). Extended receiver autonomous integrity monitoring (eRAIM) for GNSS/INS integration. J. Surv. Eng..

[23] Yang Y., He H., Xu G. (2001). Adaptively robust filtering for kinematic geodetic positioning. J. Geod..

[24] Yang C., Shi W., Chen W. (2019). Robust M–M unscented Kalman filtering for GPS/IMU navigation. J. Geod..

[25] Chang G. (2014). Robust Kalman filtering based on Mahalanobis distance as outlier judging criterion. J. Geod..

[26] Wang D., Dong Y., Li Q., Li Z., Wu J. (2018). Using Allan variance to improve stochastic modeling for accurate GNSS/INS integrated navigation. GPS Solut..

[27] Simon D. (2006). Optimal State Estimation: Kalman, H infinity, and Non- Linear Approaches.

[28] Saastamoinen J. (1972). Contributions to the theory of atmospheric refraction. Bull. Géod..

[29] El-Sheimy N., Hou H., Niu X. (2007). Analysis and Modeling of Inertial Sensors Using Allan Variance. IEEE Trans. Instrum. Meas..

